# Expression of Concern: Walach et al. The Safety of COVID-19 Vaccinations—We Should Rethink the Policy. *Vaccines* 2021, *9*, 693

**DOI:** 10.3390/vaccines9070705

**Published:** 2021-06-28

**Authors:** Vaccines Editorial Office

**Affiliations:** MDPI, St. Alban-Anlage 66, 4052 Basel, Switzerland; vaccines@mdpi.com

The journal is issuing this expression of concern to alert readers to significant concerns regarding the paper cited above [1].

Serious concerns have been raised about misinterpretation of the data and the conclusions.

The major concern is the misrepresentation of the COVID-19 vaccination efforts and misrepresentation of the data, e.g., Abstract: “For three deaths prevented by vaccination we have to accept two inflicted by vaccination”. Stating that these deaths linked to vaccination efforts is incorrect and distorted.

We will provide an update following the conclusion of our investigation. The authors have been notified about this Expression of Concern.
